# MAGT1-mediated disturbance of Mg^2+^ homeostasis lead to exhausted of HBV-infected NK and CD8^+^ T cells

**DOI:** 10.1038/s41598-017-11522-4

**Published:** 2017-10-19

**Authors:** Bo Diao, Xiaoyong Huang, Shen Guo, Chengying Yang, Guosong Liu, Yongwen Chen, Yuzhang Wu

**Affiliations:** 1 Institute of Immunology, PLA, Third Military Medical University, Chongqing, China; 2 Magceutics, Inc, 3159 Corporate Place Hayward, California, 94545 USA

## Abstract

The magnesium transporter 1 (MAGT1) is a critical regulator of basal intracellular free magnesium ([Mg^2+^]i) levels. It has been shown that MAGT1 was involved in the disorder in Mg^2+^ homeostasis after Epstein-Barr virus (EBV) infection. Here, we identified the effects of MAGT1-mediated disturbance of Mg^2+^ homeostasis on chronic hepatitis B virus (HBV)-infected natural killer (NK) and CD8^+^ T cells. The expression of MAGT1 was gradually decreased with the increase of infected time in CD8^+^ T cells, but not with that in NK cells, of the patients. Decreased level of intracellular free Mg^2+^ ([Mg^2+^]i) leads to defective expression of programmed cell death 1 (PD-1) and the NK activating receptor (NKG2D) in NK and CD8^+^ T cells. Our data illustrate that [Mg^2+^]i plays a key role in control of HBV infection.

## Introduction

Magnesium (Mg^2+^) plays an essential role, as a cofactor for ATP, nucleic acids, and numerous enzymes, in eukaryotes^1–3^. It has been reported that it serves as a second messenger in intracellular signaling. Almost 95% intracellular Mg^2+^ ([Mg^2+^]i) (10–30 mM) is complexed with ATP and other molecules in eukaryotic cells, whereas 1–5% remaining free [Mg^2+^]i (0.2–1 mM) is regulated tightly in the cytoplasm^4–6^. The magnesium transporter 1 (MAGT1) is a critical regulator of basal free [Mg^2+^]i concentrations. It conducts Mg^2+^ across the plasma membrane selectively^7,8^.

Previously, an X-linked immunodeficiency with Mg^2+^ defect, Epstein-Barr virus (EBV) infection, and neoplasia (XMEN) disease were characterized^5^. It revealed that MAGT1 was required for a T cell receptor (TCR)-stimulated Mg^2+^ influx that transiently increases free [Mg^2+^]i concentrations to temporally coordinate T cell activation^5,9,10^. Decreased free [Mg^2+^]i concentrations caused defective expression of the natural killer (NK) activating receptor (NKG2D) in NK and CD8^+^ T cells and impaired cytolytic responses against EBV. It demonstrated a link between free Mg^2+^ and antiviral immunity against EBV in humans. Mg^2+^ supplementation restored [Mg^2+^]i levels and enhanced cytotoxicity, but did not restore TCR activation in that experiment^5^.

NK cells and cytotoxic CD8^+^ T lymphocytes (CTLs) are essential for controlling viral infections and tumor immunosurveillance^6^. As a second messenger, Mg^2+^ exhibits cell-type specific activity. It has also been proved that Mg^2+^ is a kinetic regulator of signaling in T cells^5^. In the present study, we observed the effects of MAGT1-mediated disturbance of Mg^2+^ homeostasis on chronic hepatitis B virus (HBV)-infected NK and CD8^+^ T cells.

## Results

### Defects in Mg^2+^ influx after HBV-infection

**Figure 1:**
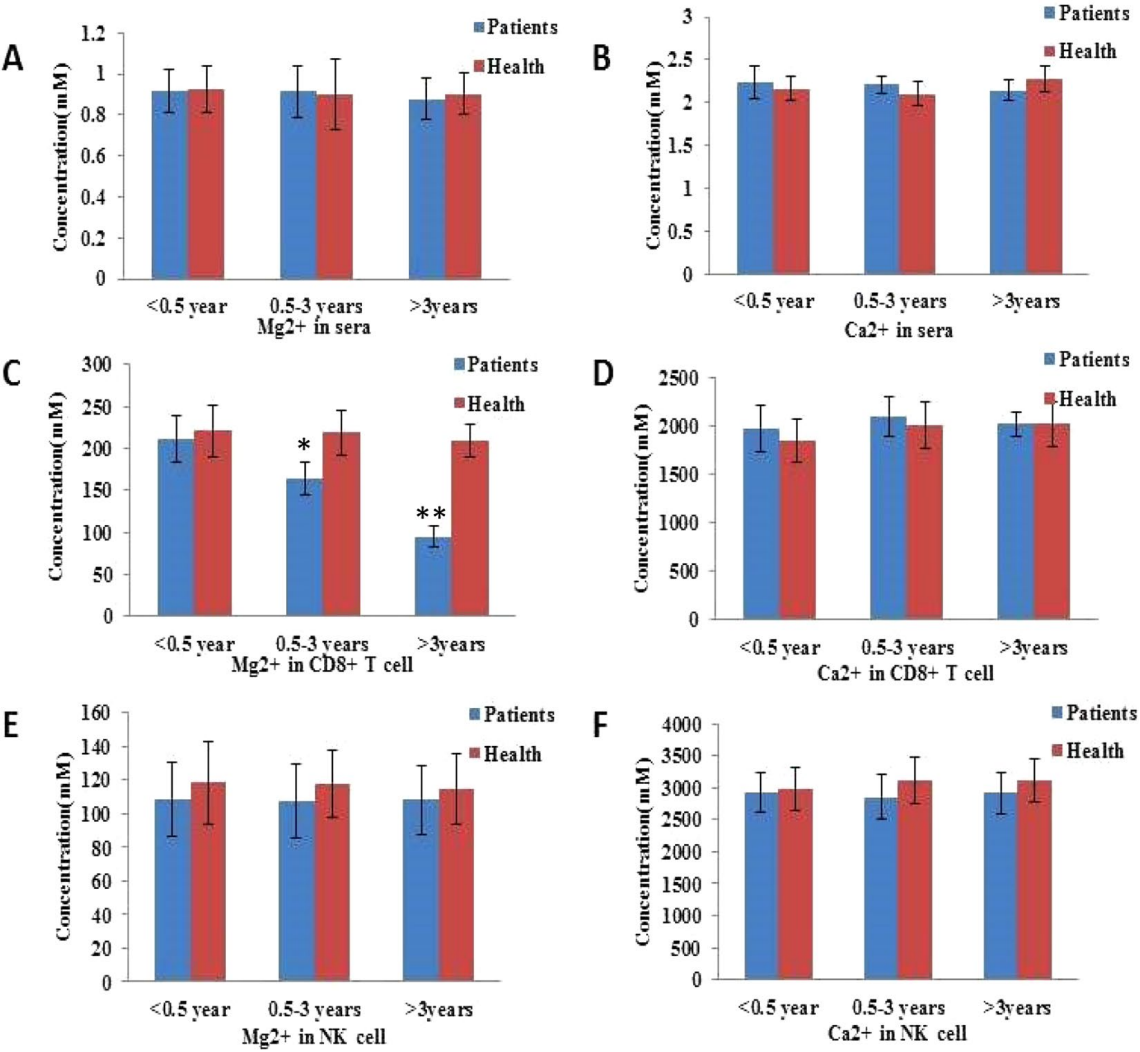
Defects in Mg^2+^ influx after HBV-infection. (**A**,**B**) The concentration of Mg^2+^ and Ca^2+^ was measured in the serum by spectrophotometry with XB-1 and indirect potentiometric determination, respectively, using a Dxc800 automatic biochemical analyzer. (**C**–**F**) The concentration of Ca^2+^ and Mg^2+^ was measured in CD8^+^ T and NK cells by the fluorescence probe Mag-Fura4-AM and Fura-2AM, respectively, using flow cytometry. Data are expressed as mean ± SD from at least 3 independent experiments, *P < 0.05 vs. Healthy controls, **P < 0.01 vs. Healthy controls.

Previous studies reported the significant role of [Mg^2+^]i in EBV control^5,9,10^. To identify the role of [Mg^2+^]i in HBV control, the concentrations of Mg^2+^ and Ca^2+^ were detected firstly. As shown in Fig. 1, the serum Mg^2+^ and Ca^2+^ concentrations were at normal levels in patients with chronic hepatitis B infection, and were similar to those in healthy controls (Fig. 1A and B). In the case of CD8^+^ T cells, Mg^2+^ levels decreased significantly in infected patients after 6 months as compared to those in non-infected patients (Fig. 1C). At the end of the 3-year investigation, Mg^2+^ levels in CD8^+^ T cells of the infected patients decreased to the lowest (Fig. 1C). Consistent with a previous study, Ca^2+^ concentration in both the groups was at normal levels (Fig. 1B and D). Moreover, the levels of both Mg^2+^ and Ca^2+^ were not significantly different in NK cells (Fig. 1E and F).

### MAGT1 expression is defective in CD8^+^ T cells of patients

**Figure 2:**
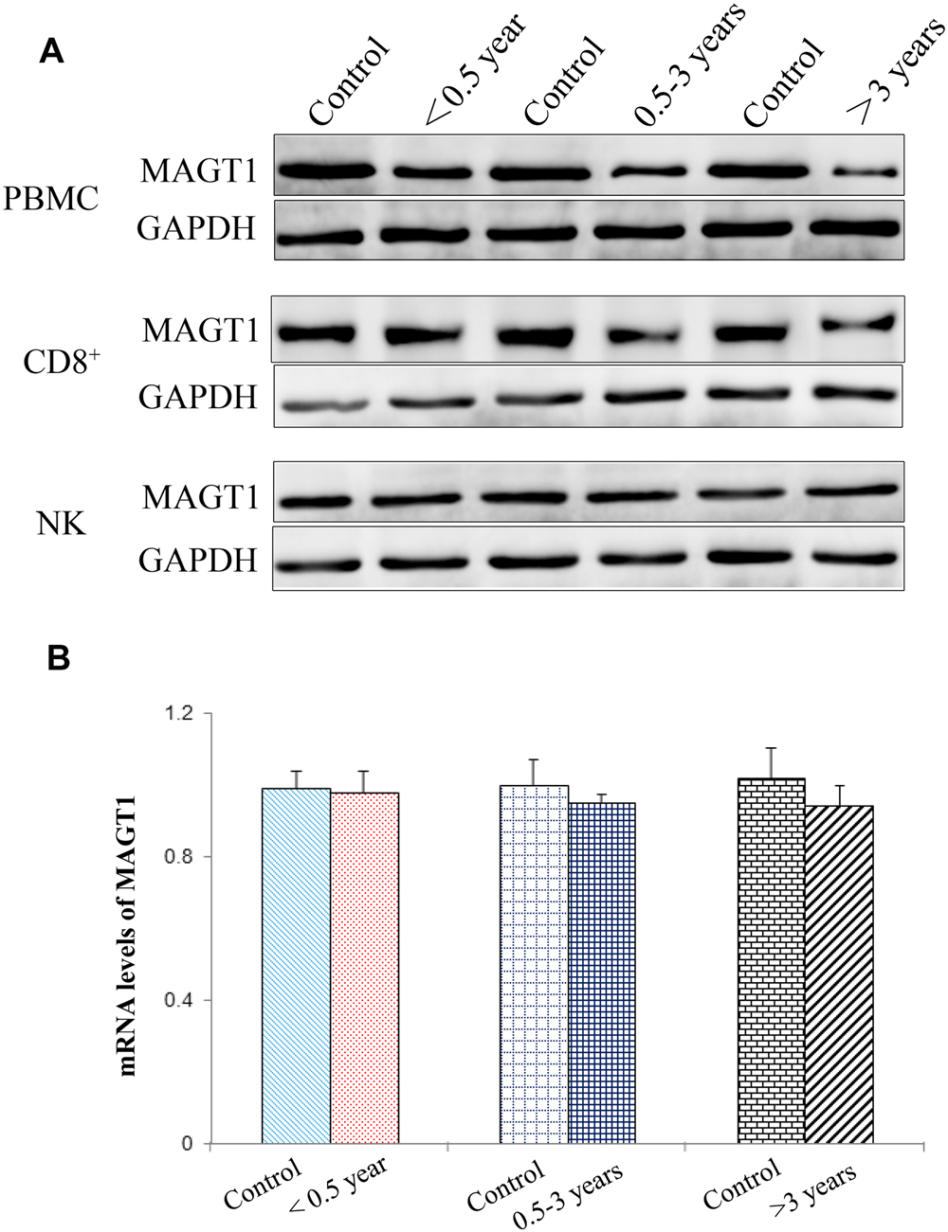
Protein level of MAGT1 is varied in CD8^+^ T cells of the patients. (**A**) Western blot analysis of MAGT1 in PBMCs and CD8^+^ and NK cells. (**B**) The mRNA level of MAGT1 in CD8^+^ cells by RT-PCR. Data are expressed mean ± SD from at least 3 independent experiments.

MAGT1 is a critical regulator of basal [Mg^2+^]i concentrations. To investigate whether defective Mg^2+^ influx is the cause of defective MAGT1 expression, MAGT1 expression was detected in different cells. As shown in Fig. 2A, MAGT1 expression was gradually decreased with the increase of infected time in CD8^+^ T cells, but not with that in NK cells, of the patients. However, the mRNA levels of MAGT1 in CD8^+^ T cells of the patients did not change as compared to those in healthy controls (Fig. 2B). These results indicated that MAGT1 expression was defective in CD8^+^ T cells of the patients possibly through post-transcriptional regulation.

To primarily confirm whether MAGT1 was regulated at the post-transcriptional level, such as through non-coding RNA, including microRNAs (miRNAs), the expression of miRNAs related to MAGT1 was detected. The detected miRNAs were selected from TargetScan (http://www.targetscan.org/vert_71/) prediction result, among which miR-199a-5p and miR-199b5p were predicted as the most conserved potential target regulators for MAGT1. As shown in Figure S1, the expression levels of miR-199a-5p and miR-199b5p in CD8^+^ T cells of the patients infected with HBV for more than 3 years were significantly increased as compared to those in normal patients.

### Expression of NKG2D and programmed cell death 1 is abnormal in CD8^+^ T cells

**Figure 3:**
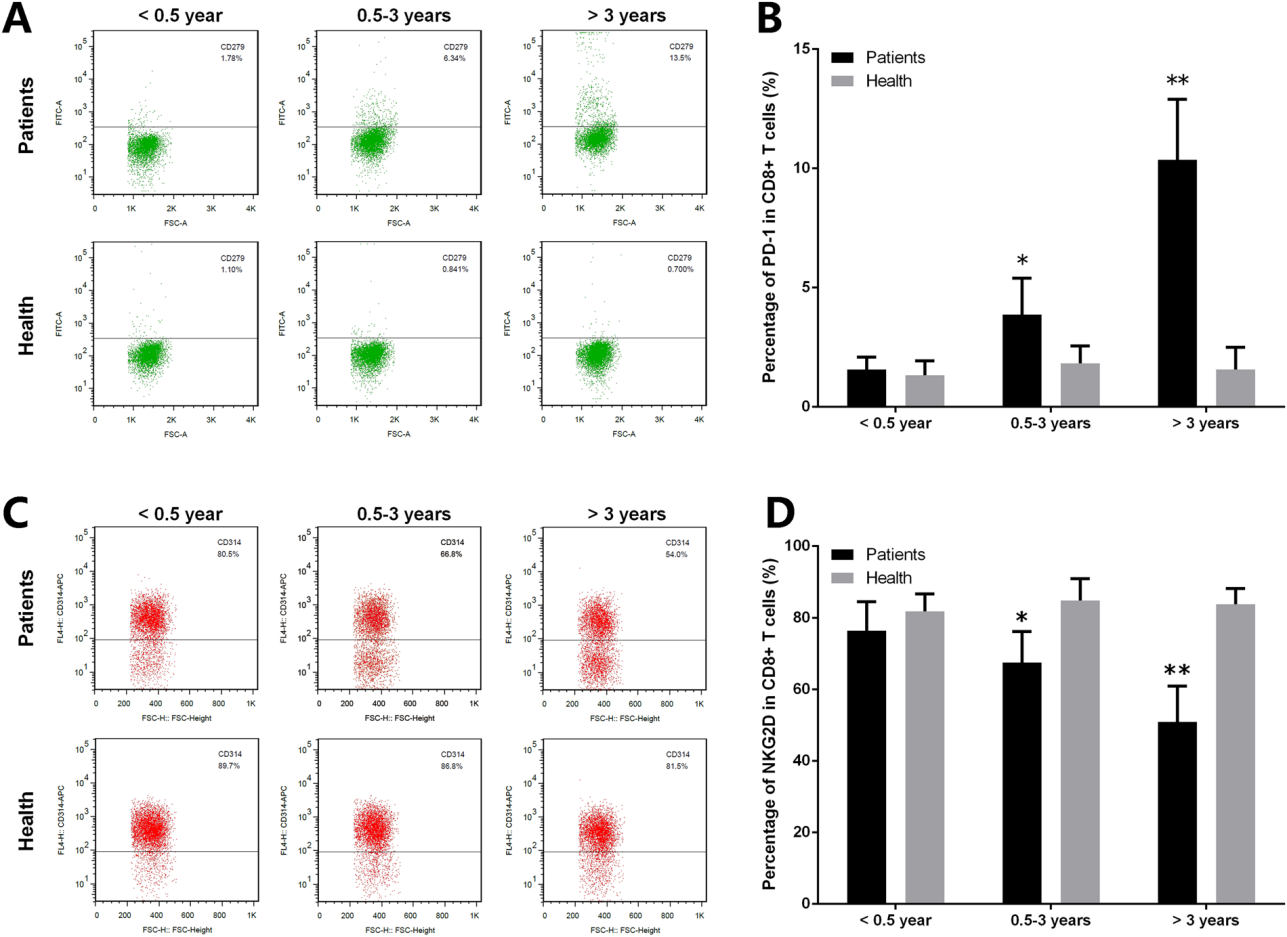
Expression of PD-1 and NKG2D is abnormal in CD8^+^ T cells. The expression level of (**A**) PD-1 and (**C**) NKG2D in CD8^+^ T cells was detected by flow cytometry. (**B**,**D**) Statistical analysis of the data obtained from flow cytometry. Data are expressed as mean ± SD from at least 3 independent experiments, *P < 0.05 vs. Healthy controls, **P < 0.01 vs. Healthy controls.

**Figure 4:**
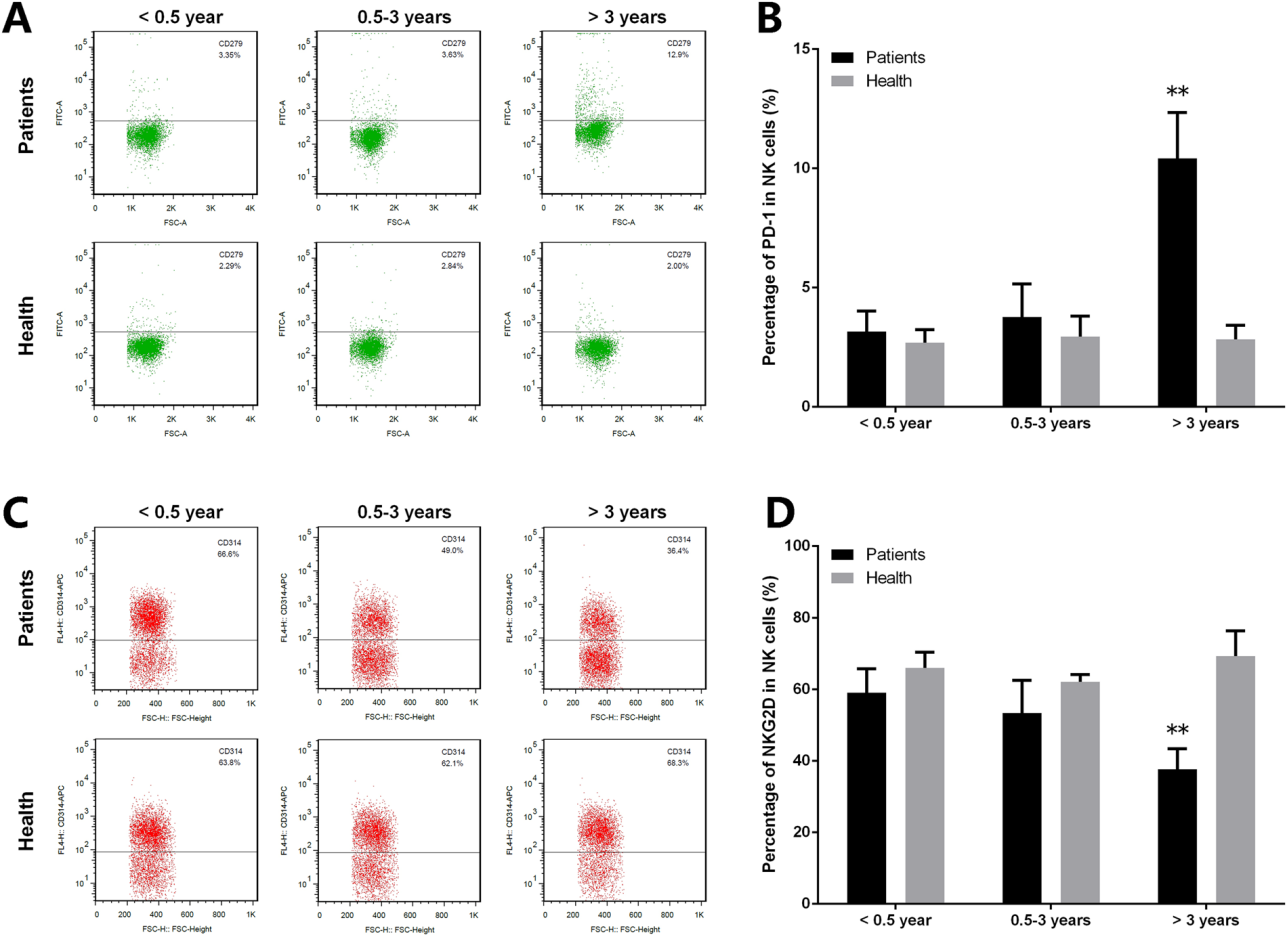
Expression of PD-1 and NKG2D in NK cells. The expression level of (**A**) PD-1 and (**C**) NKG2D in NK cells was detected by flow cytometry. (**B**,**D**) Statistical analysis of the data obtained from flow cytometry. Data are expressed as mean ± SD from at least 3 independent experiments, **P < 0.01 vs. Healthy controls.

As mentioned above, MAGT1 expression was defective after HBV infection. Lack of MAGT1 leads to decreased plasma Mg^2+^ levels in infected CD8^+^ T cells. NKG2D expression was continuously regulated by the free [Mg^2+^]i. Notably, NKG2D expression is induced by infection, cellular transformation, and cell stress in humans^11,12^. Here, the expression of NKG2D and programmed cell death 1 (PD-1) in CD8^+^ T and NK cells was detected by flow cytometry. As shown in Figs 3 and 4, the expression of NKG2D and PD-1 is abnormal in CD8^+^ T cells. NKG2D expression in CD8^+^ T cells of the patients decreased significantly in the late infection stage. On the contrary, expression of PD-1 increased significantly in CD8^+^ T cells of the patients.

### Mg^2+^ supplementation in clinical studies

**Figure 5:**
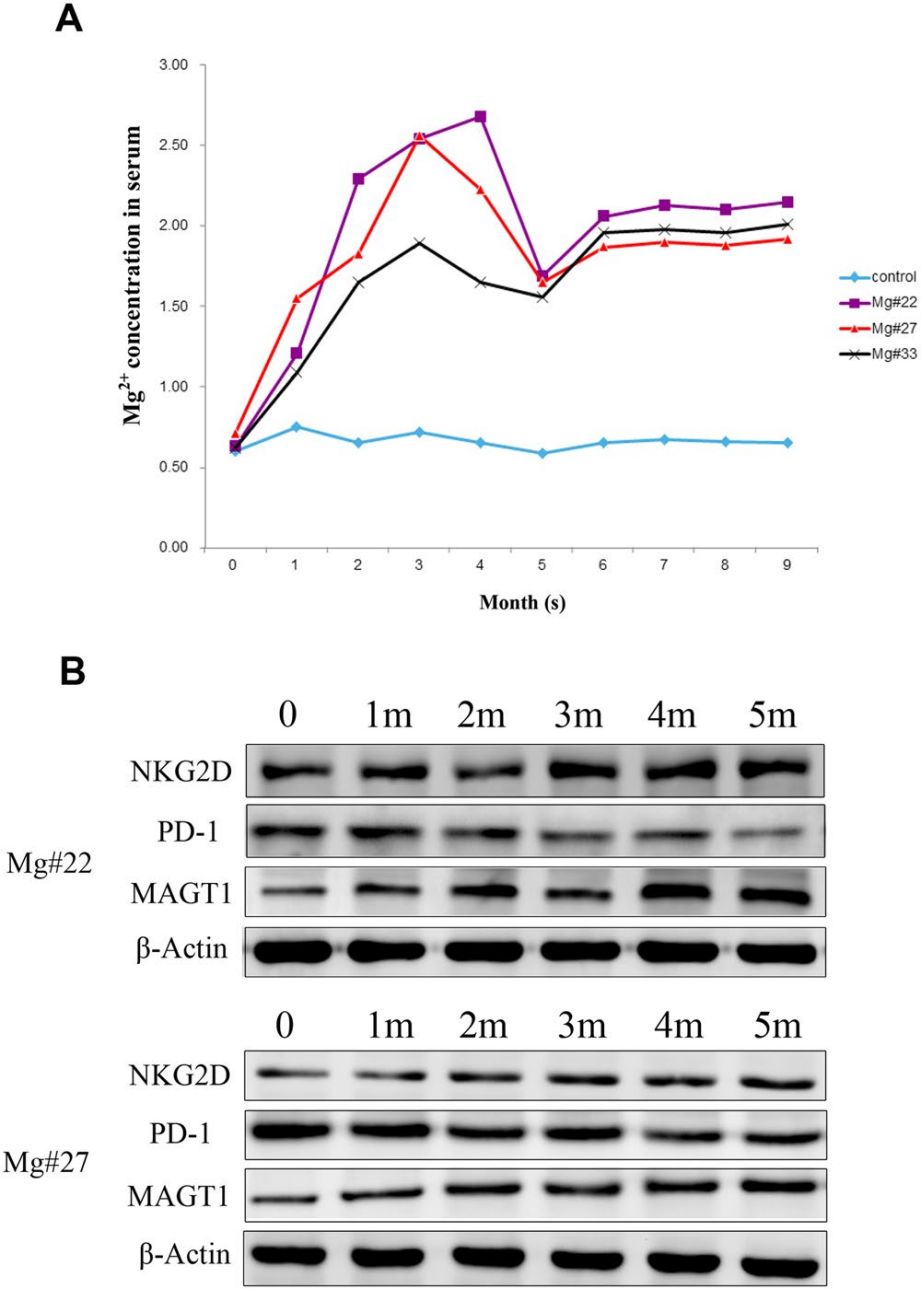
Clinical utility of Mg^2+^ supplementation. (**A**) The concentration of Mg^2+^ in the serum of Mg^2+^-supplemented patients with HBV. (**B**) The protein levels of MAGT1, PD-1, and NKG2D in the serum. Data are expressed as mean ± SD from at least 3 independent experiments.

To confirm the exact role of Mg^2+^, clinical studies were conducted. Forty patients were included in the clinical trials, which were divided into two groups—one was treated with Mg^2+^ and entecavir, while the other was treated with placebo and entecavir as the control. There was no significant difference in gender and age between the two groups. The clinical studies continued for over nine months. Consequently, the Mg^2+^ level in the serum of patients with Mg^2+^ supplementation significantly increased to an affordably normal level, and the protein level of MAGT1 also recovered to normal. In contrast, the Mg^2+^ level in the serum and the protein level of MAGT1 remained in an aberrant situation in the patients with no Mg^2+^ supplementation (Fig. 5A and B).

## Discussion

T cells are majorly acquired immune cells in humans. Depending on the CD molecules on the cell surface, T cells are divided into CD8^+^ and CD4^+^ T cells. As the major effector cells in the process of HBV infection, CD8^+^ T cells mainly exert their effects in the chronic phase and mediate immune responses (cytotoxic T lymphocyte, CTL). In this study, we found that serum Mg^2+^/Ca^2+^ concentrations were at normal levels in patients with chronic hepatitis B, and were similar to those in healthy controls. In the case of CD8^+^ T cells, the Mg^2+^ level decreased significantly in infected patients after 6 months as compared to those in non-infected patients. At the end of a 3-year investigation, Mg^2+^ levels in CD8^+^ T cells of the infected patients decreased to the lowest. Consistent with a previous study, Ca^2+^ concentration in both the groups was at normal levels^5^. The levels of both Mg^2+^ and Ca^2+^ were not significantly different in NK cells. Expression of PD-1 increased and that of NKG2D decreased in CD8^+^ T cells of the patients.

It has been reported that MAGT1 acts not only as a TCR-gated transporter, but also as a basal free [Mg^2+^] regulator^13,14^. In this study, MAGT1 expression was gradually decreased with the increase of infected time in CD8^+^ T cells, but not with that in NK cells, of the patients. The mRNA levels of MAGT1 in CD8^+^ T cells of the patients did not change as compared to those in healthy controls. The Mg^2+^ levels in CD8^+^ T cells in the plasma of patients also decreased significantly. The expression of the potential target regulators, miR-199a-5p and miR-199b-59, increased in patients with HBV, implying that decreased MAGT1 expression might be regulated at the post-transcriptional level (Figure S1).

MAGT1 expression may be defective after HBV infection. Lack of MAGT1 leads to decreased plasma Mg^2+^ levels in infected CD8^+^ T cells. NKG2D expression in CD8^+^ T cells of the patient also decreased significantly in the late infection stage. These observations are consistent with those in the previous studies^15–17^. NKG2D expression was continuously regulated by free [Mg^2+^]i. Notably, NKG2D expression is induced by infection, cellular transformation, and cell stress in humans^11,12^. Consistently, it could be explained as follows: in the late stage of chronic infection, viral replication is not well controlled and decreased Mg^2+^ level caused defective NKG2D expression. On the contrary, PD-1 expression increased significantly in CD8^+^ T cells of the patients. In the T cells, the inhibitory effect of PD-1 is based on the TCR conduction. Therefore, it might play a role as a negative regulator in CD8^+^ T cells. The clinical utility of Mg^2+^ supplementation strongly supports the data.

## Materials and Methods

### Patient samples

According to the time of HBV infection, samples from patients with chronic active HBV were divided into <0.5-year-old, 0.5–3-year-old, and >3-year-old groups. Patients in <0.5-year-old group comprised young children, who visited Wuchang District Maternal and Child Health Hospital (Wuhan, Hubei) between March and October 2014, and the HBV infection pathways studied were related to mother-to-child transmission. The 0.5–3-year-old group comprised children, who visited Wuchang District Maternal and Child Health Hospital between August and September 2014 or were recruited through kindergarten entrance health examination of Wuhan. The >3-year-old group comprised children or adult patients, who visited Wuhan General Hospital of the Chinese People’s Liberation Army (Wuhan, Hubei) between December 2013 and October 2014. There was no significant difference in gender and age between each experimental group and the control groups (Table 1). All human subjects in this study provided written informed consent in accordance with Chinese legal principles and were approved by the Medical Ethics Committee of Wuhan General Hospital of the Chinese People’s Liberation Army.

**Table 1:** Patient characteristics.

Group	n	Gender		Age (mean ± SD)
		Male	Female	
<0.5-year-old	50	24	26	(4.5 ± 0.4) months
Control 1	10	6	4	(4.2 ± 0.3) months
0.5–3-year-old	50	23	27	(4.3 ± 0.5) years
Control 2	10	5	5	(4.2 ± 0.6) years
>3-year-old	50	26	24	(31.2 ± 4.5) years
Control 3	10	4	6	(30.7 ± 3.7) years

### Cell purification and culture

PBMCs were isolated using whole blood from the normal control and patients by Ficoll-Paque PLUS (GE Healthcare, USA) and density-gradient centrifugation. The cells were washed twice in phosphate buffered saline (PBS), resuspended at a density of 10^6^ cells/mL in complete RPMI 1640 medium (Lonza, Switzerland) supplemented with 10% fetal bovine serum (FBS), 2 mM glutamine, and penicillin and streptomycin (100 U/mL each; Invitrogen, USA), and incubated in a humidified incubator at 37 °C and 5% CO_2_.

### Isolation and identification of CD8^+^ T and NK cells

To prepare NK cells, PBMCs were incubated with a cocktail of anti-CD3, anti-CD19, and anti-CD14 monoclonal antibody (mAb)-coated microbeads, and NK cells were isolated by passing the PBMCs through a magnetic cell separation system (MZSC; Miltenyi Biotec, Germany) with column type VR. More than 95% of the cells were confirmed to be CD56^+^ (Miltenyi Biotech, Germany) NK cells by flow cytometry. Simultaneously, to isolate CD8^+^ T cells, PBMCs were suspended in labeling buffer and incubated with anti-CD8 mAb-coated microbeads. CD8^+^ T cells were isolated using a magnetic cell separation system with column type VR. More than 95% of the cells were confirmed to be CD8^+^ (Miltenyi Biotech, Germany) T cells by flow cytometry.

### Measurement of Mg^2+^ and Ca^2+^

The concentration of Ca^2+^ and Mg^2+^ in the serum was measured by spectrophotometry with XB-1 and indirect potentiometric determination, respectively, using a Dxc800 automatic biochemical analyzer (Beckman, USA). The cell density was adjusted to 1.5 × 10^6^ cell/mL in assay buffer without Mg^2+^ and Ca^2+^, and cells were respectively loaded with 3 μM Mag-Fura4-AM (ThermoFisher, USA) or 2 μM Fura-2AM (ThermoFisher, USA) for 20 min at room temperature (RT) in the dark. Ten milliliters PBS was added, centrifuged at 300 × *g* for 5 min, and the cells were resuspended in assay buffer. The measurement of [Mg^2+^]i and [Ca^2+^]i were conducted at RT using flow cytometry. Flow cytometry data were analyzed using FlowJo software 3.3.

### Analysis of the expression of PD-1 and NKG2D by flow cytometry

CD8^+^ T and NK cells were resuspended at a density of 1 × 10^5^ cells/mL, and 20 μL APC anti-human NKG2D (BD, USA) or FITC anti-human PD-1(BD, USA) was added. After mixing, the cells were stained for 30 min under dark conditions at RT. Stained cells were immediately detected using a flow cytometer, and data were analyzed using FlowJo software 3.3.

### RT-PCR for MAGT1, miR-199a-5p, and miR-199b-5p quantification

Total RNA was extracted from CD8^+^ T cells using TRIzol reagent (Invitrogen, CA, USA). The first-strand cDNA synthesis was carried out in a 20-µL reaction mixture containing 1 µg DNase-treated total RNA using the superscript III reverse transcriptase (Promega, WI, USA, http://www.promega.com) and random primers (Promega) according to the manufacturer’s instructions, and 2 ng cDNA products were used in each RT-PCR reaction. Primer information is as follows: *MAGT1*, forward: 5′-AGACACTGGAG TACTGGAAAT-3′ and reverse: 5′-TGACAGGAGAATCGCTTAAAC -3′. *GAPDH* served as the internal control, forward: 5′-TTCTATA AATTGAGCCCG CAG-3′ and reverse: 5′-CGATACCAA AGTTGTCATGGA-3′. The sequence of the miR-199a-5p, RT primer: 5′-GTCGTATCCAGTGCGTGTCGTG GAGTCGGCAATTGCACTGG ATACGACGAACAG-3′, forward: 5′-GGGCCCAGTGTT CAGACTAC-3′, reverse: 5′-CAGTGCGTGTCGTGGAGT-3′. The sequence of the miR-199b-5p, RT primer: 5′-GTCGTATCCAGTGCGTGTCGTGGAGTCGGCAATTGCACTG GATACGAC GAACAG-3′, forward: 5′-GGGCCCAGTGTTTAGACTAT-3′, reverse: 5′-CAGT GCGTGTCGTGGAGT-3′. U6 served as the internal control: RT primer: 5′-AACGC TTCACGAATTTGCGT-3′, forward: 5′-CTCGCTTCGGCAGCACA-3′, reverse: 5′-AACGCT TCACGAATTTGCGT-3′. The relative expression ratio of MAGT1, miR-199a-5p, and miR-199b-5p was calculated by the 2^−△△CT^ method.

### Immunoblotting

The cells were washed in PBS and immediately lysed using cell lysis buffer containing 1% Triton X-100, 1% NP40, 50 mM Tris-Cl pH 8, 150 mM NaCl, 2 mM EDTA, 1 mM Na_3_VO_4_, 1 mM NaF, phosphatase inhibitor cocktail (Sigma), and complete protease inhibitor cocktail (Roche). Protein concentration was quantified by BCA assay (Bio-Rad). Equal amounts of protein were resolved on an SDS-PAGE gel and then transferred onto nitrocellulose membranes (Millipore, Bedford, USA). After blocking in 5% skimmed milk for 30 min, the membranes were incubated with primary antibodies, such as anti-MAGT1 (Abcam, 1:1000), anti-PD-1 (Abcam, 1:1000), anti-NKG2D (Abcam, 1:1000), anti-β-actin (Abcam, 1:1000), and anti-GAPDH (CST, 1:1000), at 4 °C overnight. After washing, the membrane was incubated with corresponding horseradish peroxidase-conjugated secondary antibodies (Abcam, Cambridge, USA; 1:1000) at RT for 1 h. The signal was detected using enhanced chemiluminescence system (Millipore). The band intensity was analyzed with Image J software (NIH, USA) and the protein levels were normalized by GAPDH or β-actin.

### Statistical analysis

Data are expressed as the means ± SD from at least 3 independent experiments. Differences between groups were analyzed by Student’s *t*-test and one-way ANOVA with Bonferroni correction for the comparison between three groups, the level of significance was set at *P* < 0.05. The analyses were performed using SPSS 16.0 software (SPSS Inc., Chicago, IL, USA).

## Electronic supplementary material

Supplementary Information

## References

[1] Cakmak I, Kirkby EA (2008). Role of magnesium in carbon partitioning and alleviating photooxidative damage. Physiol Plantarum.

[2] Cowan JA (2002). Structural and catalytic chemistry of magnesium-dependent enzymes. Biometals.

[3] Yang W, Lee JY, Nowotny M (2006). Making and Breaking Nucleic Acids: Two-Mg^2+^-Ion Catalysis and Substrate Specificity. Mol Cell.

[4] Grubbs RD, Maguire ME (1987). Magnesium as a regulatory cation: criteria and evaluation. Magnesium.

[5] Li FY (2011). Second messenger role for Mg^2+^ revealed by human T-cell immunodeficiency. Nature.

[6] Orange JS (2002). Human natural killer cell deficiencies and susceptibility to infection. Microbes Infect.

[7] Quamme GA (2010). Molecular identification of ancient and modern mammalian magnesium transporters. Am J Physiol Cell Physiol.

[8] Zhou H, Clapham DE (2009). Mammalian MAGT1 and TUSC3 are required for cellular magnesium uptake and vertebrate embryonic development. Proc Natl Acad Sci USA.

[9] Chaigne-Delalande B (2013). Mg^2+^ Regulates Cytotoxic Functions of NK and CD8 T Cells in Chronic EBV Infection Through NKG2D. Science.

[10] Li FY, Lenardo MJ, Chaigne-Delalande B (2011). Loss of MAGT1 abrogates the Mg^2+^ flux required for T cell signaling and leads to a novel human primary immunodeficiency. Magnes Res.

[11] Ogasawara K, Lanier LL (2005). NKG2D in NK and T Cell-Mediated Immunity. J Clin Immunol.

[12] Zhang B (2012). Immune surveillance and therapy of lymphomas driven by Epstein-Barr virus protein LMP1 in a mouse model. Cell.

[13] Deason-Towne F, Perraud AL, Schmitz C (2011). The Mg^2+^ transporter MAGT1 partially rescues cell growth and Mg^2+^ uptake in cells lacking the channel-kinase. FEBS Lett.

[14] Schweigel M, Kolisek M, Nikolic Z, Kuzinski J (2008). Expression and functional activity of the Na/Mg exchanger, TRPM7 and MAGT1 are changed to regulate Mg homeostasis and transport in rumen epithelial cells. Magnes Res.

[15] Chemnitz JM, Parry RV, Nichols KE, June CH, Riley JL (2004). SHP-1 and SHP-2 associate with immunoreceptor tyrosine-based switch motif of programmed death 1 upon primary human T cell stimulation, but only receptor ligation prevents T cell activation. J Immunol.

[16] Nishimura H, Nose M, Hiai H, Minato N, Honjo T (1999). Development of Lupus-like Autoimmune Diseases by Disruption of the PD-1 Gene encoding an ITIM motif-carrying immunoreceptor. Immunity.

[17] Nishimura H (2001). Autoimmune dilated cardiomyopathy in PD-1 receptor-deficient mice. Science.

