# Supplementary material for: MAGT1-mediated disturbance of Mg2+ homeostasis lead to exhausted of HBV-infected NK and CD8+ T cells
Source: Sci Rep. 2017 Oct 19;7:13594. doi: 10.1038/s41598-017-11522-4 (PMC5648775; doi:10.1038/s41598-017-11522-4)
Supplement: Supplementary file 1 — Supplementary Information [file 41598_2017_11522_MOESM1_ESM.docx]

**ONLINE DATA SUPPLEMENT**

**MAGT1-mediated disturbance of Mg^2+^ homeostasis lead to exhausted of HBV-infected NK and CD8^+^ T cells**

Bo Diao^1,^ Xiaoyong Huang^1,^ Shen Guo ^1,^ Chengying Yang^1,^ Guosong Liu^2,^ Yongwen Chen^1^ & Yuzhang Wu^1, *^

^1^ Institute of Immunology, PLA, Third Military Medical University, Chongqing, China

^2^ Magceutics, Inc, 3159 Corporate Place Hayward, California 94545, USA

^*^ Author for correspondence.

Dr. Yuzhang Wu, Institute of Immunology, PLA, Third Military Medical University, Chongqing, 400038, China.

E-mail: wuyuzhang@tmmu.edu.cn

Dr. Yongwen Chen, Institute of Immunology, PLA, Third Military Medical University, Chongqing, 400038, China.

E-mail: [yongwench@163.com](mailto:yongwench@163.com)

**Supplementary** **Figure S1.** The expression levels of miRNAs related to MAGT1 in patients with HBV.





**Figure S1.** The expression levels of miRNAs related to MAGT1 in patients with HBV. Quantitative real-time PCR analysis of expression level of miR-199a-5p and miR-199b-5p in CD8^+^ T cells of patients with HBV infection for greater-than 3 years, and the CD8+ T cells of healthy people were used as a control. Data are expressed as mean ± SD from at least 3 independent experiments, ^**^*P*< 0.01 vs. Healthy controls, ^***^*P*< 0.001 vs. Healthy controls.
